# Extraction condition optimization and effects of drying methods on physicochemical properties and antioxidant activities of polysaccharides from *Astragalus cicer* L.

**DOI:** 10.1038/s41598-018-21295-z

**Published:** 2018-02-20

**Authors:** Hongmei Shang, Menghan Wang, Ran Li, Mengying Duan, Hongxin Wu, Haizhu Zhou

**Affiliations:** 1 College of Animal Science and Technology, Jilin Agricultural University, Changchun, 130118 China; 2 Key Laboratory of Animal Nutrition and Feed Science of Jilin Province, Changchun, 130118 China; 3 Key Laboratory of Animal Production, Product Quality and Security, Ministry of Education, Changchun, 130118 China; 4 Grassland Research Institute of CAAS, Hohhot, 010010 China

## Abstract

Response surface methodology (RSM) including three variables was performed to optimize the extraction parameters of *Astragalus cicer* L. polysaccharides (ACPs). The influence of different drying techniques on the physicochemical properties and antioxidant abilities of ACPs were evaluated. The ACPs were dried with hot air (HD), vacuum (VD) and freeze drying (FD) methods. The optimal conditions for ACPs extraction were as follows: water to raw material ratio of 25 mL/g, extraction time of 61 min and temperature of 75 °C. Under these parameters, an ACPs yield of 10.97% was obtained. HPLC analysis showed that the monosaccharide compositions of the three ACPs dried with HD, VD or FD techniques were identical. The three ACPs exhibited antioxidant abilities in a concentration-dependent manner. ACPs dried with the FD method (FD-ACPs) had the best antioxidant activities, which might be related to their smaller molecular weight and higher uronic acid content. At the determined concentration of 1 mg/mL, the ferric reducing power, and DPPH and ABTS free radical scavenging capacities of FD-ACPs were 0.762, 75.30% and 99.21%, respectively. Therefore, FD was a good choice for the drying of *Astragalus cicer* L. polysaccharides.

## Introduction

*Astragalus* L., the largest genus in the family of *Leguminosae* with approximately 2500–3000 species, is widely distributed throughout the temperate regions of the world^1^. Several species of *Astragalus* L. are considered to have potential anhidrotic, diuresis, antidote and tonic effects^2^. Certain species of *Astragalus* L. are also used for the treatment of nephritides, mellitus, leucocythemia and metrocarcinoma^3^. Many studies have been carried out using compounds such as flavones, saponins, alkaloids, polysaccharides, sterols or phenols from various species of *Astragalus* L^4^.

Polysaccharides, a type of carbohydrate polymer macromolecule, are important bioactive constituents in plants. A number of biological functions of polysaccharides, such as antioxidant, antivirus, immunoregulation, hypoglycemic activity and blood fat reduction have been reported^5,6^. The development and utilization of *Astragalus cicer* L. are both connected with the extraction efficiency of polysaccharides from its tissue. Nevertheless, there have not yet been studies regarding the extraction conditions of *Astragalus cicer* L. polysaccharides (ACPs). In general, water extraction technology is the most common method used for extracting polysaccharides from plant tissue because of its safety and low cost. However, the various conditions of water extraction technologies can lead to differences in yield, purity, physicochemical properties and biological activities of the extracted polysaccharides. Response surface methodology (RSM) is a commonly used procedure for optimizing the extraction parameters of polysaccharides. One type of RSM, the Box-Behnken Design (BBD), has been widely used in the optimization of the extraction procedure because it dramatically reduces the number of experimental trails, saving time and decreasing expenses.

Drying techniques have significant effects on the physicochemical characteristics and bioactivities of the resulting polysaccharides^7^. Hot air drying (HD), freeze drying (FD) and vacuum drying (VD) are commonly used techniques for drying polysaccharides. Each technique has advantages, such as the easy accessibility and low cost of HD, the vacuum environment, which prevents the oxidation reaction of polysaccharides during VD, and the vacuum and freeze conditions, which protect the biological activities of polysaccharides during FD. However, significant changes of physicochemical properties and activities of polysaccharides may appear when using the high temperature of HD and VD^8^. One disadvantage of FD is the higher energy consumption during the drying process due to refrigeration and deoxygenation conditions. At present, there are no available data regarding the selection of drying methods of ACPs.

This study was therefore designed to optimize the extraction conditions of ACPs using the RSM methodology and to determine the physicochemical characteristics and antioxidant properties of ACPs as influenced by the different drying techniques. The physicochemical characteristics of the ACPs investigated were chemical composition, moisture content, pH, relative viscosity, solubility, molecular weight and monosaccharide composition. The antioxidant properties of ACPs were evaluated based on the determination of scavenging activities against DPPH and ABTS free radicals, as well as ferric reducing power.

## Results and Discussion

### Effect of water to raw material ratio on ACPs yield

**Figure 1:**
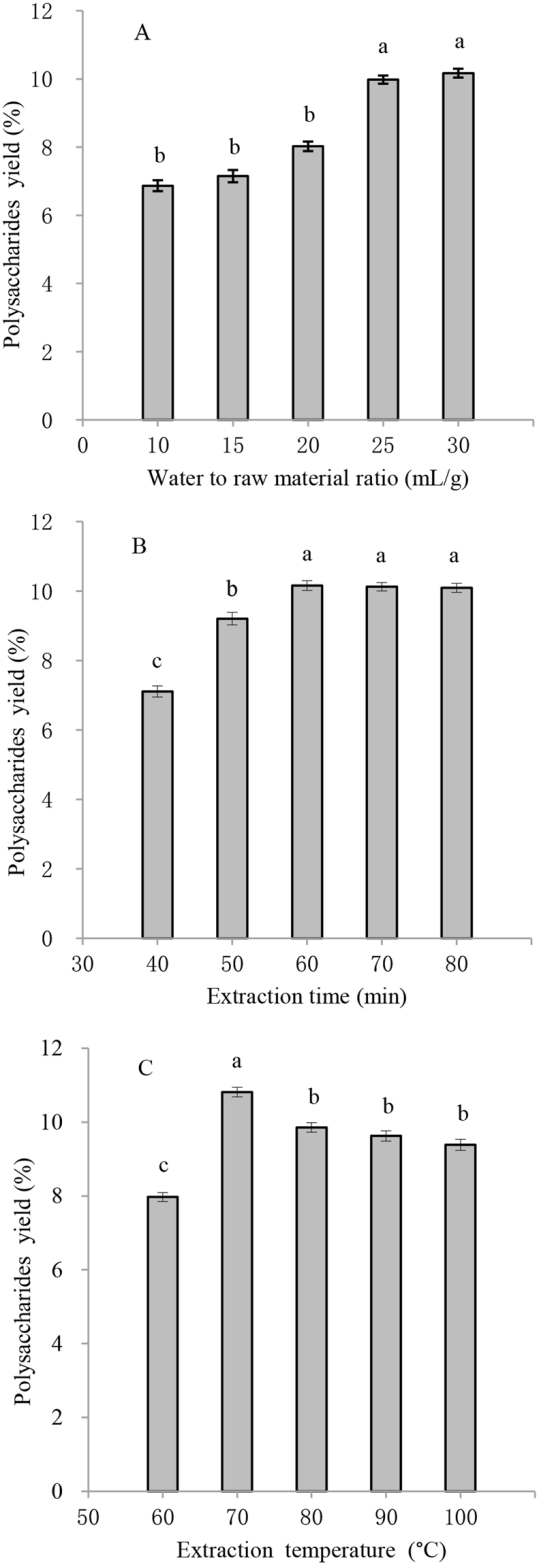
Influences of water to raw material ratio **(A)**, extraction time **(B)** and extraction temperature **(C)** on the extraction yield of *Astragalus cicer* L. polysaccharides. Bar charts with different lowercase letters significantly differ (*P* < 0.05).

The ACPs yield from different water to raw material ratios from 10 to 30 mL/g is shown in Fig. 1A. As the water to raw material ratio increased from 10 to 25 mL/g, the ACPs yield increased from 6.87% to 9.98% (*P* < 0.05). However, the ACPs yield did not significantly increase at the water to raw material ratio of 30 mL/g compared to 25 mL/g (*P* > 0.05). The reason for this might be due to the concentration difference between the *Astragalus cicer* L. tissue and the extraction solvent^9^. The solution amount from *Astragalus cicer* L. tissue increased with the water to raw material ratio increase. Nevertheless, with a further increase in the water to raw material ratio, the amount of extracted ACPs did not significantly improve because of the increasing diffusion distance of ACPs from plant tissue^10^. Therefore, 25 mL/g was used as the water to raw material ratio in subsequent RSM experiments.

### Effect of extraction time on ACPs yield

Extraction time is one of the main factors to optimize for polysaccharide extraction conditions^11^. The ACPs yield from different extraction times ranging from 40 to 80 min is shown in Fig. 1B. As the extraction time increased from 40 to 60 min, the ACPs yield increased from 7.11% to 10.16% (*P* < 0.05). However, a further lengthening of extraction time (70 and 80 min) resulted in a slight reduction in ACPs yield compared with the extraction time of 60 min. This phenomenon might be explained by the degradation of ACPs during a long extraction time at high temperatures^12^. Therefore, an extraction time of between 50 and 70 min was used in subsequent RSM experiments.

### Effect of extraction temperature on ACPs yield

Extraction temperature is another routine factor that requires optimization in the extraction of polysaccharides. The ACPs yield from different extraction temperatures (60 to 100 °C) is presented in Fig. 1C. A significant increase (from 7.97% to 10.81%) in the extraction yield of ACPs was found when the extracting temperature was in the range of 60 to 70 °C (*P* < 0.05). However, a further increase in temperature (80, 90 and 100 °C) caused a significant reduction in ACPs yield compared with the extraction temperature at 70 °C (*P* < 0.05). This might be due to an increased capacity of the solvent to solubilize the polysaccharides from *Astragalus cicer* L. tissue, with a concomitant decrease in the viscosity of the sample solution when the temperature rose, improving the ACPs yield. However, the ACPs structure might be destroyed when using too high of an extraction temperature^13^. Therefore, the temperature range of 60 to 80 °C was selected for use in subsequent RSM experiments.

### Box-Behnken design of ACPs extraction

BBD is useful for RSM because it is based on single factor experiments and provides a scale of experimental variables and avoids excessive preliminary experiments. According to the single-factor experiment results, 17 runs of three variables were performed to determine the optimum extraction conditions for ACPs. The three variables were water to raw material ratio encoded as *X*_1_, extraction time encoded as *X*_2_ and extraction temperature encoded as *X*_3_. The designed experimental parameters and ACPs yields (from 7.24% to 10.85%) are shown in Table 1. By performing a multiple regression analysis of the experimental values, equation (1) of the response model for ACPs yield (*Y)* was obtained as follows:

1 $$\begin{array}{rcl}Y( \% ) & = & -117.4098+3.0452{X}_{1}+1.3517{X}_{2}+1.2941{X}_{3}+1\mathrm{.5987E}-\mathrm{003}{X}_{1}{X}_{2}\\  &  & -{\rm{2}}\mathrm{.9236E} \mbox{-} \mathrm{003}{X}_{1}{X}_{3}+5.5594{\rm{E}}-004{X}_{2}{X}_{3}-0.0576{{X}_{1}}^{2}-0.0118{{X}_{2}}^{2}-8.3502{\rm{E}}-003{{X}_{3}}^{2}\end{array}$$

**Table 1:** The Box-Behnken design matrix and the results of the ACPs yields.

Run	Water to raw material ratio (*X*_1_) (mL/g)	Extraction time (*X*_2_) (min)	Extraction temperature (*X*_3_) (°C)	ACPs yields (%)	
				Actual value	Predicted value
1	−1 (20)	−1 (50)	0 (70)	7.75	7.76
2	1 (30)	−1 (50)	0 (70)	8.21	8.19
3	−1 (20)	1 (70)	0 (70)	7.96	7.98
4	1 (30)	1 (70)	0 (70)	8.73	8.72
5	−1 (20)	0 (60)	−1 (60)	7.24	7.21
6	1 (30)	0 (60)	−1 (60)	8.09	8.09
7	−1 (20)	0 (60)	1 (80)	9.21	9.21
8	1 (30)	0 (60)	1 (80)	9.47	9.50
9	0 (25)	−1 (50)	−1 (60)	7.76	7.78
10	0 (25)	1 (70)	−1 (60)	8.04	8.05
11	0 (25)	−1 (50)	1 (80)	9.39	9.38
12	0 (25)	1 (70)	1 (80)	9.88	9.86
13	0 (25)	0 (60)	0 (70)	10.85	10.78
14	0 (25)	0 (60)	0 (70)	10.80	10.78
15	0 (25)	0 (60)	0 (70)	10.79	10.78
16	0 (25)	0 (60)	0 (70)	10.82	10.78
17	0 (25)	0 (60)	0 (70)	10.64	10.78

The ANOVA analysis of the response surface model for ACPs extraction is presented in Table 2. The regression model was significant (*P* < 0.0001), and the lack of fit was insignificant (*P* = 0.9043), indicating that the model was adequate to predict the ACPs yield. The determination coefficient (*R*^2^) of 0.9988 suggested that only 0.12% of the variation could not be explained by the regression model. As presented in Table 2, the cross-product coefficient of *X*_1_ *X* _2_ was significant (*P* < 0.05). The linear coefficients of *X*_1_, *X*_2_ and *X*_3_, cross-product coefficient of *X*_1_ *X* _3,_ and quadratic coefficients of *X*_1_^2^, *X*_2_^2^ and *X*_3_^2^ were highly significant (*P* < 0.01). The other term coefficient (*X*_2_ *X* _3_) was not significant (*P* > 0.05). Therefore, *X*_1_, *X*_2_ and *X*_3_ designed in this study were significant parameters that influenced ACPs yield.

**Table 2:** ANOVA for response surface quadratic model of ACPs extraction.

Source	Sum of squares	df	Mean square	*F*-value	*P*-value
Model	26.32	9	2.92	647.57	<0.0001**
*X*_1_	0.67	1	0.67	149.35	<0.0001**
*X*_2_	0.28	1	0.28	62.45	<0.0001**
*X*_3_	5.83	1	5.83	1290.75	<0.0001**
*X*_1_X_2_	0.026	1	0.026	5.66	0.0490*
*X*_1_X_3_	0.085	1	0.085	18.92	0.0034**
*X*_2_X_3_	0.012	1	0.012	2.74	0.1420
*X*_1_^2^	8.72	1	8.72	1931.03	<0.0001**
*X*_2_^2^	5.83	1	5.83	1290.41	<0.0001**
*X*_3_^2^	2.94	1	2.94	650.03	<0.0001**
Residual	0.032	7	4.516E-003		
Lack of fit	3.773E-003	3	1.258E-003	0.18	0.9043
Pure error	0.028	4	6.961E-003		
Cor Total	26.35	16			
*R* ^2^	0.9988				
Adj *R*^2^	0.9973				
C.V%	0.73				

^*^P values < 0.05 were considered to be significant. ^**^P values < 0.01 were considered to be highly significant.

**Figure 2:**
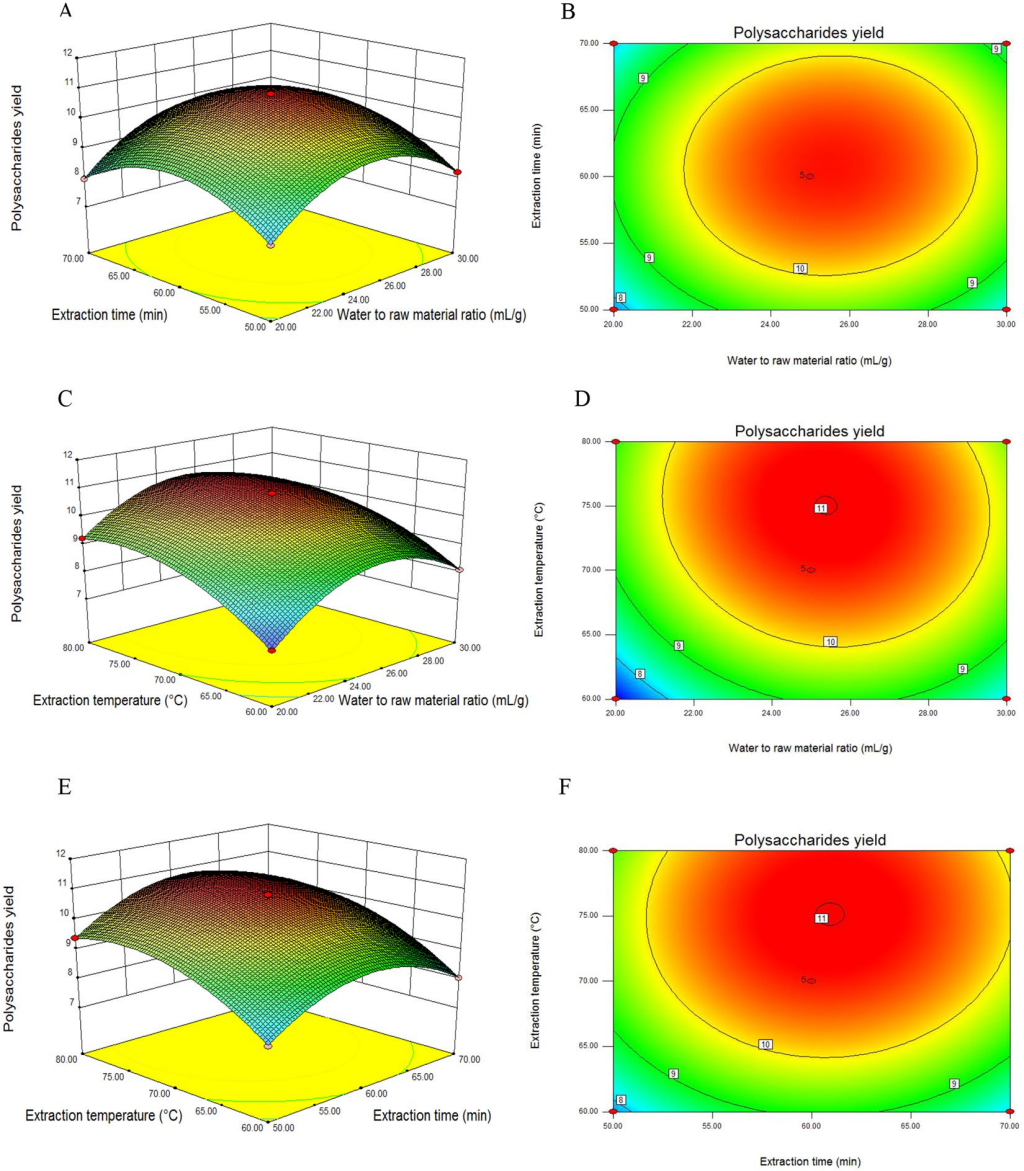
3D response surface **(A**,**C** and **E)** and 2D contour **(B**,**D** and **F)** plots exhibiting the influences of the variables on the yield of *Astragalus cicer* L. polysaccharides.

Response surface plots are used to visually evaluate the interactions of variables on the ACPs yield^14^. The pattern of the 2-dimensional (2D) contour plot (elliptical, selliform or circular) indicates the different interactions between two variables. Elliptical or selliform 2D plots indicate significant interactions between two variables, while circular 2D plots indicate non-significant interactions between two variables. As shown in Fig. 2, the 2D plot of *X*_1_ and *X*_2_ (Fig. 2B) was elliptical, suggesting that the interaction between these two variables is significant (*P* < 0.05). A similar significance for the interaction between *X*_1_ and *X*_3_ is shown in Fig. 2D (P < 0.05). The 2D plot of *X*_2_ and *X*_3_ (Fig. 2F) was circular, suggesting that the interaction between these two variables is non-significant (*P* > 0.05).

It could be concluded from the regression model that the optimum extraction conditions of ACPs were *X*_1_ = 25.39 mL/g, *X*_2_ = 60.95 min, and *X*_3_ = 75.07 °C. The predicted ACPs yield was 11.02%. To facilitate convenient operation, *X*_1_ = 25 mL/g, *X*_2_ = 61 min, and *X*_3_ = 75 °C were used for ACPs extraction. The verification experiment was performed, and the actual ACPs yield was found to be 10.97 ± 0.13% (*n* = 3).

### Effects of drying techniques on physicochemical characteristics of ACPs

The yield and activities of polysaccharides are related to dehydration methods and temperature, because polysaccharides from different plant sources possess different denaturation temperatures. In this study, ACPs were dried using three different techniques (HD, VD and FD). ACPs dried with HD method were termed HD-ACPs, VD-ACPs were obtained by VD technology, and ACPs dried with FD method were termed FD-ACPs. The activities of polysaccharides are influenced by many characteristics, such as their chemical composition, monosaccharide composition and molecular weight^15^. Therefore, these parameters were determined for the ACPs obtained by the three drying methods in this study.

The chemical composition of ACPs dried using the three methods is listed in Table 3. The yields of the ACPs were 10.65% for FD-ACPs, 9.75% for HD-ACPs and 9.91% for VD-ACPs. The total polysaccharides content of HD-ACPs, VD-ACPs and FD-ACPs were 52.48%, 55.43% and 58.90%, respectively. The protein content of HD-ACPs, VD-ACPs and FD-ACPs were 0.88%, 0.83% and 0.91% with a uronic acid content of 2.49%, 2.52% and 2.71%, respectively. Good correlation between the uronic acid content and the antioxidant ability of tea polysaccharides was reported by Chen *et al*.^16^. FD-ACPs possessed the highest uronic acid content among the three dried ACPs, which might be related to the higher temperature in the HD and VD conditions.

**Table 3:** The chemical composition of ACPs dried with three methods.

Samples	HD-ACPs	VD-ACPs	FD-ACPs
Polysaccharide yield (%)	9.75 ± 0.07^b^	9.91 ± 0.07^b^	10.65 ± 0.10^a^
Total polysaccharide content (%)	52.48 ± 0.44^c^	55.43 ± 0.80^b^	58.90 ± 0.97^a^
Protein content (%)	0.88 ± 0.01^a^	0.83 ± 0.07^a^	0.91 ± 0.04^a^
Uronic acid content (%)	2.49 ± 0.13^b^	2.52 ± 0.03^b^	2.71 ± 0.09^a^
Moisture content (%)	8.13 ± 0.24^a^	8.27 ± 0.35^a^	8.33 ± 0.17^a^
pH	7.19 ± 0.04^a^	7.17 ± 0.02^a^	7.22 ± 0.03^a^
Relative viscosity	1.01 ± 0.01^b^	1.09 ± 0.01^a^	1.13 ± 0.05^a^

The data is shown as the mean ± standard deviation (n = 3). Different superscripts in the same row indicate significant difference (*P* < 0.05). HD-ACPs dried by hot air drying method; VD-ACPs dried by vacuum drying method; FD-ACPs dried by freeze drying method.

**Figure 3:**
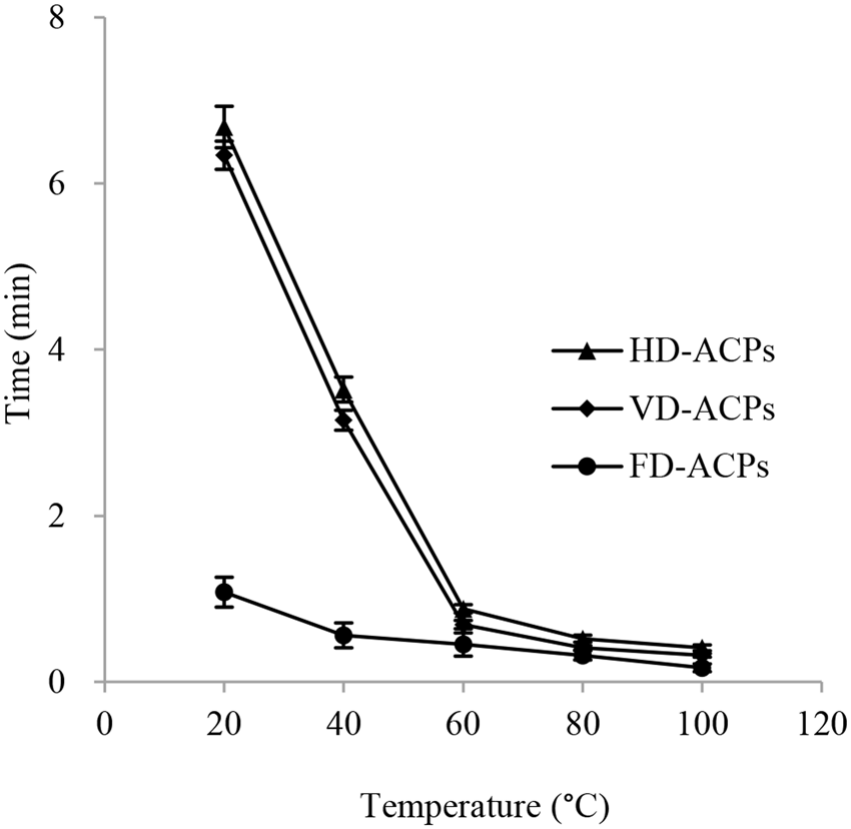
Solubility of polysaccharides from *Astragalus cicer* L. dried by three methods: HD-ACPs dried by hot air drying method; VD-ACPs dried by vacuum drying method; FD-ACPs dried by freeze drying method.

Moisture content is an important property of polysaccharides. As shown in Table 3, the moisture content of HD-ACPs, VD-ACPs and FD-ACPs was 8.13%, 8.27% and 8.33%, respectively (*P* > 0.05). Low moisture content suggests that ACPs have the potential to be widely used in the food processing field. The pH of HD-ACPs, VD-ACPs and FD-ACPs was determined to be 7.19, 7.17 and 7.22, respectively (*P* > 0.05). The relative viscosities (to deionized water) of HD-ACPs, VD-ACPs and FD-ACPs increased from 1.01 to 1.13. As the temperature increased, the time for ACPs to dissolve gradually decreased (Fig. 3). ACPs dried with the freeze method showed the shortest dissolving time, which might be related to the loose structure of FD-ACPs.

**Figure 4:**
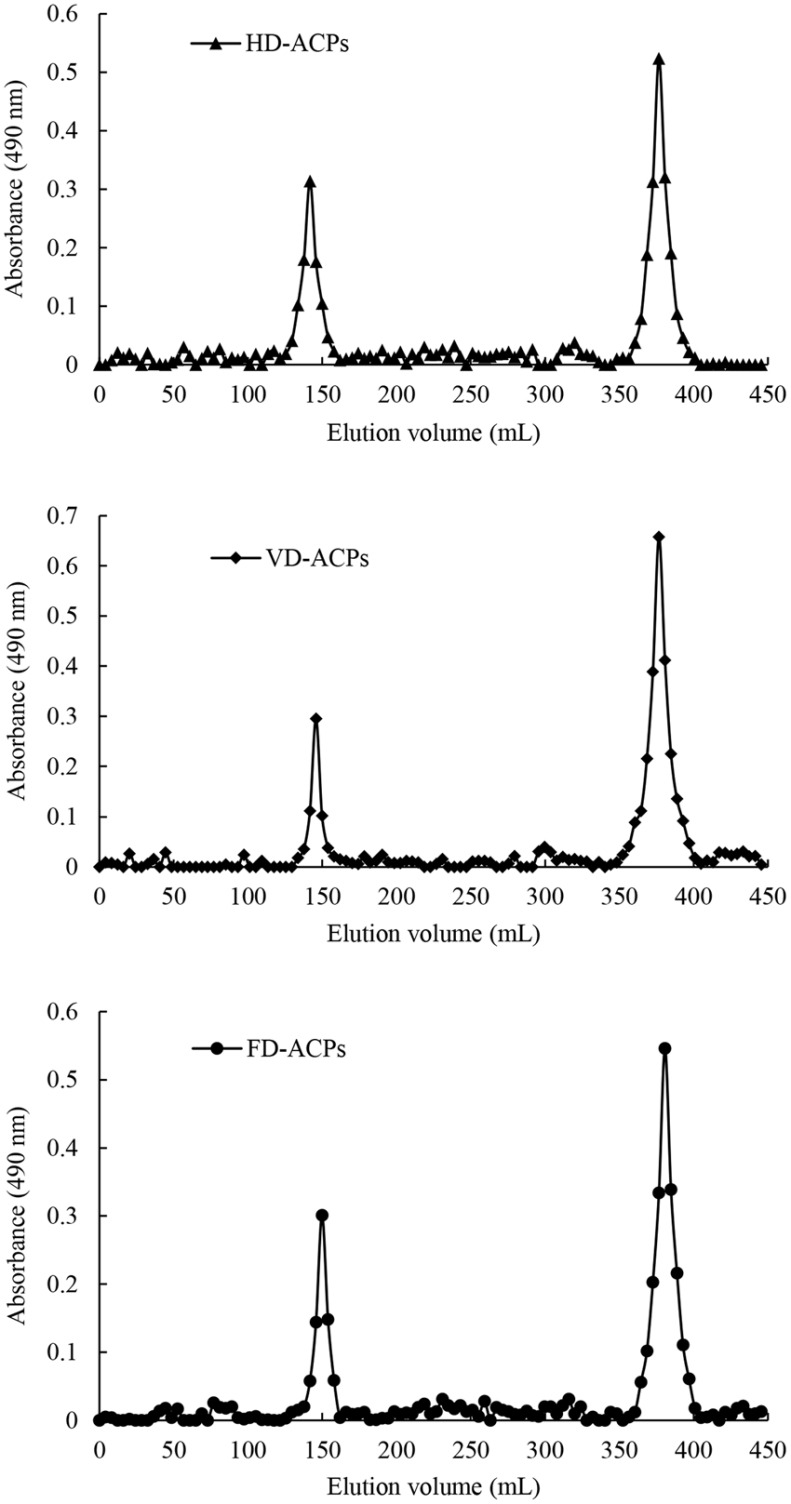
Molecular weight distribution of polysaccharides from *Astragalus cicer* L. dried by three methods: HD-ACPs dried by hot air drying method; VD-ACPs dried by vacuum drying method; FD-ACPs dried by freeze drying method.

In general, the molecular weights of polysaccharides extracted from plants are highly dispersed. The molecular weight distribution of the ACPs is presented in Fig. 4. When the ACPs were dried by hot air, two distinct groups were found, possessing the molecular weights of 356.95 × 10^4^ Da and 2243.48 Da. VD-ACPs also showed two distinct groups with molecular weights of 314.34 × 10^4^ Da and 2243.48 Da. When ACPs were freeze dried, the molecular weight distribution showed two distinct groups with the molecular weights of 276.82 × 10^4^ Da and 1975.70 Da. The molecular weights of the three ACPs obtained by HD, VD, and FD techniques were different, which might be related to the intermolecular aggregation of ACPs during different drying processes. VD-ACPs and HD-ACPs showed higher molecular weights, which suggests that the polysaccharides molecules aggregate easily at relative high temperature. A portion of the hydration layer was removed during the drying process, which destroyed the polysaccharide structure and resulted in aggregation^17^. The molecular weight of FD-ACPs was smaller than that of VD-ACPs or HD-ACPs. Generally, polysaccharides with smaller molecular weights show better activities compared to the polysaccharides with larger molecular weights, which might be due to the polysaccharides with lower molecular weights being able to pass through biological membranes without the restriction of the immune system in the body^18^.

The monosaccharide analysis showed that HD-ACPs, VD-ACPs and FD-ACPs were composed of rhamnose, galacturonic acid, glucose, galactose and arabinose. The molar ratios of the five monosaccharides in HD-ACPs were 1.22, 1.00, 11.98, 10.48 and 3.62. VD-ACPs consisted of the five monosaccharides with the molar ratios of 1.12, 1.00, 11.71, 10.75 and 3.56. FD-ACPs were composed of the five monosaccharides with the molar ratios of 1.22, 1.00, 11.64, 10.51 and 3.74. The monosaccharide analysis results indicated the homogeneity of the three ACPs.

### Effects of drying techniques on antioxidant activities of ACPs

Many different *in vitro* methods have been used to determine the antioxidant activities of polysaccharides. The DPPH free radical is one kind of stable radical being used for assessing the antioxidant properties of polysaccharides^19^. The DPPH free radical scavenging activity assay is based on the reduction of DPPH ethanol solution in the presence of a hydrogen donating antioxidant, resulting in the formation of the non-radical form of DPPH-H. The assay can accommodate many samples in a short period and is sensitive enough to detect active ingredients at low concentrations^20^. ABTS radicals can react with antioxidants through the acceptance of a hydrogen atom or an electron^18^. The ABTS radical scavenging activity assay was shown to be simple and quick, and has been extensively used to evaluate the antioxidant activity of biological samples^7^. The ion Fe^3+^ can activate the reaction of lipid peroxidation in the human body^21^. Ferric reducing power serves as a significant potential index of antioxidant activity. In the reaction system, the addition of the antioxidant substance reduces the Fe^3+^ in the potassium ferricyanide to the Fe^2+^ form and is monitored by the formation of Prussian Blue at 700 nm. Therefore, the ferric reducing power can directly reflect the donation of electrons or hydrogen, and has been widely used to investigate the antioxidant activities of natural compounds^18^.

**Figure 5:**
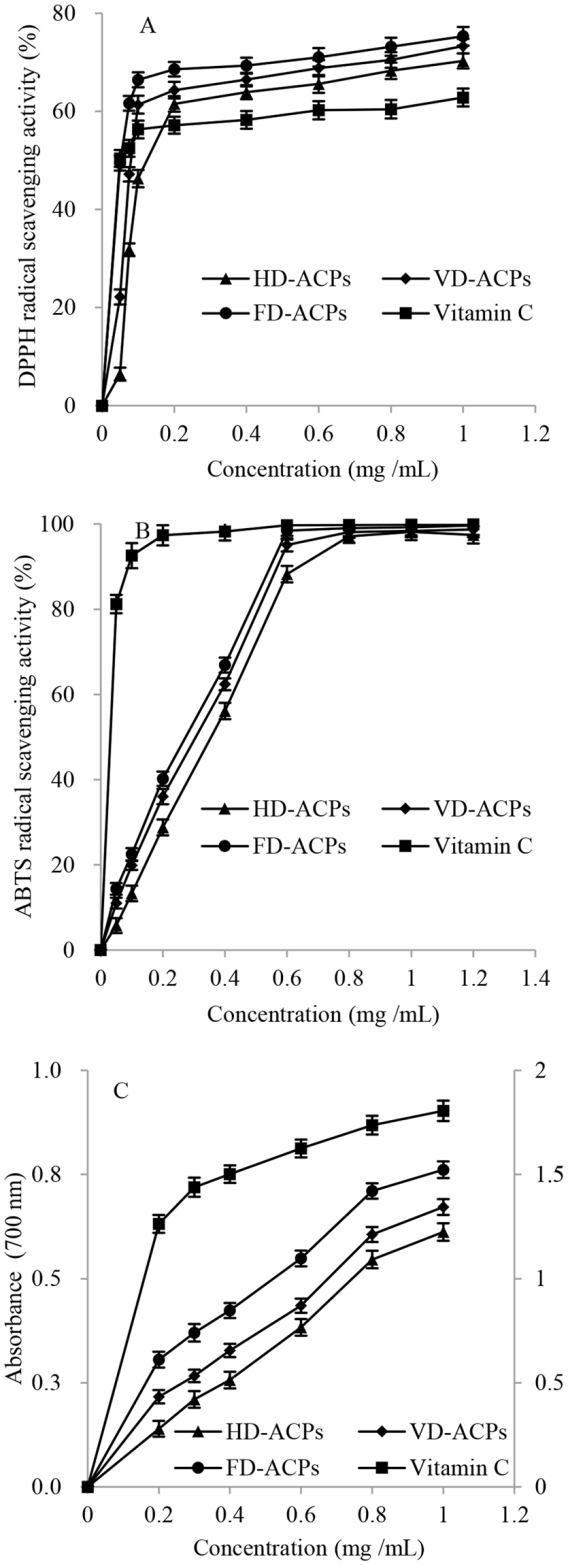
Antioxidant activities of *Astragalus cicer* L. polysaccharides dried by three methods: HD-ACPs, dried by hot air drying method; VD-ACPs dried by vacuum drying method; FD-ACPs dried by freeze drying method.

In Fig. 5A, the DPPH radical scavenging abilities of the three ACPs can be found in a quadratic concentration-dependent pattern (*P* < 0.05). FD-ACPs showed stronger DPPH radical scavenging activities compared to HD-ACPs or VD-ACPs (*P* < 0.05) at each concentration from 0.05 to 1 mg/mL. This might be related to differences in chemical composition, such as total polysaccharides and uronic acid content among the three dried ACPs. The scavenging activities were 70.27%, 73.30% and 75.30% for HD-ACPs, VD-ACPs and FD-ACPs, respectively, at a concentration of 1 mg/mL. ACPs (from 0.2 to 1 mg/mL) dried with the three techniques showed higher scavenging activities than vitamin C (*P* < 0.05), suggesting that ACPs may be donors of electrons or hydrogen to eliminate the free radical DPPH.

As shown in Fig. 5B, the ABTS radical scavenging power of FD-ACPs was stronger than ACPs dried with either the HD or VD methods (*P* < 0.05). Hydroxyl groups are important for polysaccharides to exhibit ABTS radical scavenging activity^18^. Nevertheless, oxidation of hydroxyl groups occurred under the presence of oxygen during hot air drying. Therefore, HD-ACPs showed lower ABTS radical scavenging power compared to the other two ACPs. FD-ACPs (1 mg/mL) had an ABTS scavenging activity of 99.21 ± 2.33%, and the scavenging abilities of the three ACPs (1 mg/mL) reached the scavenging levels of vitamin C (*P* < 0.05).

As shown in Fig. 5C, ACPs dried with the three methods presented a nicely linear relationship to the determination concentration (*R*^2^ > 0.99, *P* < 0.05) in their ferric reducing powers. Ferric reducing powers of all the ACPs increased with elevated concentration, which suggests that ACPs could terminate the radical chain reactions. FD-ACPs showed stronger ferric reducing powers compared to HD-ACPs or VD-ACPs (*P* < 0.05) in each determination concentration from 0.2 to 1 mg/mL. The ferric reducing powers of ACPs were significantly lower compared to vitamin C (*P* < 0.05). FD-ACPs (1 mg/mL) had a reducing power of 0.762. Nevertheless, garlic^22^ and *Hohenbuehelia serotina*^18^ polysaccharides (10 mg/mL) showed a reducing power of approximately 0.5. The ferric reducing powers of polysaccharides were related to their molecular weights^23^. The polysaccharides with smaller molecular weights showed better ferric reducing powers than the polysaccharides with larger molecular weights due to the more exposed reducing ends in the smaller polysaccharides^24^. Therefore, the high ferric reducing power of FD-ACPs might be related to its lower molecular weight among the three ACPs.

The antioxidant activity of polysaccharides was associated with their chemical composition, monosaccharide composition and molecular weight^25^. The higher antioxidant activity of FD-ACPs might be due to its higher content of uronic acid and lower molecular weight. In the polysaccharides, the existence of uronic acid groups could trigger the hydrogen atom of the anomeric carbon^26^. It has been reported that the higher the content of galacturonic acid, the stronger the antioxidant activity of the polysaccharides^27^. According to correlation analysis, a higher uronic acid content and lower molecular weight correlated to a higher antioxidant activity of polysaccharides from *Dioscorea hemsleyi*^28^. The lower molecular weight of *Grifola frondosa* polysaccharides extracted by combined enzyme enzymolysis rather than by boiling water played an important role in their higher antioxidant activities^29^. An explanation for this molecular weight-antioxidant activity relationship is that, at an equal mass concentration, lower molecular weight polysaccharides may provide a larger number of hydroxyl groups for antioxidant action^12^. The low molecular weight polysaccharides have more opportunities to come into contact with free radicals because of their increased water solubility and larger surface area^30^.

In conclusion, RSM was performed to obtain ACPs extracting parameters. The ACPs yield was significantly affected by different variables (*P* < 0.05). The optimal conditions in which to extract ACPs were determined to be a water to raw material ratio of 25 mL/g, an extraction time of 61 min and an extraction temperature of 75 °C, affording an ACPs yield of 10.97%. The three ACPs dried with different methods showed homogeneity in the monosaccharide composition. FD-ACPs had the highest solubility, the lowest molecular weight and the highest antioxidant activity. The high antioxidant activity of FD-ACPs might be related to its higher content of uronic acid and lower molecular weight. Therefore, the freeze-drying method was found to be the preferred technology to dry *Astragalus cicer* L. polysaccharides. Further work is essential to determine the antioxidant activities *in vivo* and the potential antioxidant mechanism of ACPs.

## Materials and Methods

### Plant and chemicals

Astragalus cicer L. in bloom stage was obtained from Jilin Agricultural University (Changchun, Jilin, China). The plant materials were dehydrated (50 °C) and crushed into powders (1 mm). The lipids of the powders were removed by soaking in ethanol (85%, v/v) for one day. The insoluble substance was dehydrated at 50 °C and prepared for polysaccharide extraction. DPPH, ABTS, vitamin C and monosaccharide standard were obtained from Sigma (St. Louis, MO, USA). Other chemicals were analytically pure reagents.

### ACPs extraction

ACPs were extracted by distilled water based on the designed conditions. The extract was centrifuged for 15 min (3000 rpm), then one-quarter volume of the primary supernatant was obtained by vacuum concentration at 60 °C. The starch fraction in the concentrated solution was removed by α-amylase at 60 °C. Four times the volume of ethanol was mixed with the concentrated extraction solution to precipitate the ACPs (4 °C, 12 h). Subsequently, the precipitates were obtained by centrifugation at 3000 rpm for 15 min, and washed successively with ether, absolute ethanol and acetone. The extracts were dissolved with distilled water and protein was removed with the Sevag reagent (chloroform: normal butanol, 4:1, v/v) method. After dialysis (MWCO 1400 Da, Union Carbide) of the extraction solution, the ACPs were obtained by freeze drying. The ACPs yield was calculated with the following equation (2):

2 $${\rm{ACPs}}\,{\rm{yield}}\,( \% )=\frac{{W}_{{\rm{ACPs}}}}{{W}_{{\rm{sample}}}}\times 100$$

where *W*_ACPs_ and *W*_sample_ are the weights of ACPs and *Astragalus cicer* L. powder used, respectively.

### Design of Box-Behnken

A single-factor experiment was first designed to evaluate the variable range for ACPs extraction. Each *Astragalus cicer* L. powder material was extracted with deionized water based on the designed variables including *X*_1_, *X*_2_ and *X*_3_ according to the ACPs extraction process mentioned above. The BBD of ACPs yield (*Y*) was then carried out. As shown in Table 1, the design was made up of 17 experimental runs. The three levels of each variable were coded as −1, 0 and + 1. A second-order polynomial mode (3) was used to optimize the extraction conditions of the ACPs as follows:

3 $$Y={A}_{0}+\sum _{i=1}^{3}{A}_{i}{X}_{i}+\sum _{i=1}^{3}{A}_{ii}{X}_{i}^{2}+\sum _{i=1}^{2}\sum _{j=i+1}^{3}{A}_{ij}{X}_{i}{X}_{j}$$

where *Y* is the dependent variable (ACPs yield); *X*_*ij*_ is the interaction term; *X*_*i*_^*2*^ is the quadratic term; *A*_0_, *A*_*i*_, *A*_*ii*_ and *A*_*ij*_ represent the coefficients of the intercept term, the linear term, the quadratic term, and the interaction term of two variables, respectively; and *X*_*i*_ and *X*_*j*_ are variables.

### Drying process of ACPs

ACPs were extracted under the optimum conditions according to the BBD. The ACPs extracts were dehydrated with three different techniques (HD, VD and FD) until the weights became constant. HD was performed using a drying oven (101–2-BS, Shanghai Yuejin Medical Instrument Co., LTD, Shanghai, China) at 50 °C, and the dried polysaccharides were termed HD-ACPs. VD was carried out in a vacuum drying chamber (DZF, Shanghai Longyue Instrument Equipment Co., LTD, Shanghai, China) at 50 °C, and the dried polysaccharides were termed VD-ACPs. FD was carried out in a lyophilizer (SCIENTZ-12N, Ningbo Scientz Biotechnology Co., LTD, Ningbo, China) at −70 °C, and the resulting polysaccharides were termed FD-ACPs.

### Physicochemical properties of ACPs

Chemical composition parameters, including the total polysaccharide, uronic acid and protein content of the ACPs were analyzed using colorimetry methods. The total polysaccharide content in the ACPs was determined with the phenol-sulfate method^31^. The uronic acid content was determined with the *m*-hydroxybiphenyl method^32^, and protein content was determined using Bradford’s method^33^. The moisture content was determined based on the report of Kong *et al*.^34^.

A pH meter was used to determine the pH values of the ACPs at the concentration of 2 mg/mL. The relative viscosity of ACPs (10 mg/mL) to deionized water was determined using a rotation viscometer (NDJ-8S, Shanghai Jitai Electronic Technology Co., LTD, Shanghai, China) at 25 °C. The solubility of ACPs was measured at 20, 40, 60, 80, and 100 °C according to the method as described by Shang *et al*.^7^.

Before determining the molecular weight distribution of the polysaccharides obtained by the three drying methods, the ACPs samples were initially purified by using a DEAE-52 cellulose column (3.5 cm × 20 cm), and eluted first with deionized water, then with a linear gradient (0 to 1 mol/L) of NaCl at a flow rate of 2 mL/min. The fractions (8 mL/tube) were collected automatically, and the polysaccharide content of each tube was determined with the phenol-sulfate method^31^. Fractions (one fraction eluting with distilled water) containing carbohydrates were collected and concentrated to measure the molecular weight of ACPs by gel filtration chromatography on a Sepharose CL-6B column (2.6 cm × 100 cm). A series of dextrans (T-10, T-40, T-70, T-500) were used as calibration standards. The eluent was deionized water (0.9 mL/min), and 4.05 mL fractions were collected per tube. The polysaccharide content of each tube was determined using the phenol-sulfate method^31^.

The monosaccharide composition of the ACPs obtained by the three drying methods was determined using high-performance liquid chromatographic (HPLC) by following methods as described previously^35^ with minor modifications. ACPs samples (2 mg) were hydrolyzed with 0.5 mL trifluoroacetic acid (TFA, 2 mol/L) in a sealed flask filled with N_2_ at 120 °C for 2 h. After hydrolysis, the excess TFA in the system was removed by repeated co-evaporation with ethanol at 45 °C. The dry hydrolysate samples of ACPs or monosaccharide standards were then added to a 0.5 mL methanol solution of 1-phenyl-3-methyl-5-pyrazolone (PMP, 0.5 mol/L) and 0.5 mL NaOH (0.3 mol/L) for derivatization at 70 °C for 30 min. The mixed solution was then centrifuged (10 000 rpm) for 5 min. The supernatant was mixed with 0.05 mL HCl (0.3 mol/L), and the reaction mixture was extracted with chloroform to remove excess PMP. The aqueous layer was filtered with a 0.22 μm membrane and used for analysis of the monosaccharide composition of ACPs by a Shimadzu 2010AHT HPLC system (SHIMADZU, Kyoto, Japan). The HPLC system was equipped with a UV detector (245 nm) and an Amethyst C18 column (4.6 mm × 250 mm, 5 μm, Sepax, Delaware, USA). The mobile phase was a mixture of phosphate buffered saline (PBS, 0.1 mol/L, pH 7) and acetonitrile (80:20, v/v) with a flow rate of 1 mL/min. The column was kept at 25 °C, and the injection volume was 10 μL.

### Measurement of antioxidant activities of ACPs

The DPPH radical scavenging activity of ACPs was assessed based on a literature procedure with some modifications^36^. In brief, the DPPH solution (0.1 mM in ethanol, 1 mL) was added into 3 mL ACPs solution (dissolved in distilled water) at different concentrations (0.05, 0.075, 0.1, 0.2, 0.4, 0.6, 0.8 and 1.0 mg/mL). The mixture was kept in the dark for 30 min, and then the absorbance of the reaction mixture was determined at 517 nm. Vitamin C served as the positive control. The scavenging activity of ACPs against DPPH radical was evaluated by the followed equation (4):

4 $${\rm{DPPH}}\,{\rm{radical}}\,{\rm{scavenging}}\,{\rm{activity}}\,{\rm{of}}\,{\rm{ACPs}}\,( \% )=\frac{[{A}_{0}-({A}_{1}-{A}_{2})]\times 100}{{A}_{0}}$$

where *A*_0_ is the control absorbance (water instead of the ACPs solution); *A*_1_ is the absorbance of the ACPs; and *A*_2_ is the absorbance of the combined reaction reagents, except that the DPPH solution is replaced with ethanol.

The scavenging activity of ACPs against ABTS radical was evaluated with a literature method with some modifications^37^. Vitamin C served as the positive control. The ABTS radical cation was produced by the reaction between ABTS solution (5 mL, 7 mM) and K_2_S_2_O_8_ aqueous solution (1 mL, 15 mM) for 12 h in the dark. The ABTS radical cation solution was then diluted with deionized water to obtain an absorbance of 0.70 ± 0.02 at 734 nm. The ABTS radical cation solution (3 mL) was added into 0.75 mL ACPs solution (dissolved in distilled water) at different concentrations (0.05, 0.1, 0.2, 0.4, 0.6, 0.8, 1.0 and 1.2 mg/mL). After reaction for 15 min, the absorbance at 734 nm was determined. The scavenging activity of ACPs against ABTS radical was evaluated by the followed equation (5):

5 $${\rm{ABTS}}\,{\rm{radical}}\,{\rm{scavenging}}\,{\rm{activity}}\,{\rm{of}}\,{\rm{ACPs}}\,( \% )=\frac{[{A}_{0}-({A}_{1}-{A}_{2})]\times 100}{{A}_{0}}$$

where *A*_0_ is the absorbance of the control (ACPs solution was replaced with water); *A*_1_ is the absorbance of the ACPs solution; and *A*_2_ is the absorbance of all the reaction reagents, except that the ABTS solution is replaced with distilled water.

The ferric reducing power of ACPs was determined according to procedures included in the literature^38,39^. Vitamin C was used as the positive control. ACPs in distilled water (1.5 mL) were mixed with sodium phosphate buffer (1.5 mL, 0.2 M, pH 6.6) and potassium ferricyanide solution (1.5 mL, 1%, w/v). After incubation at 50 °C for 20 min, the mixture was quickly cooled. Trichloroacetic acid (1.5 mL, 10%, w/v) was added to the mixture, and the mixture was centrifuged at 3000 rpm for 10 min. The supernatant (1.5 mL) was mixed with deionized water (1.5 mL) and ferric chloride (0.3 mL, 0.1%, w/v). After 10 min, the absorbance was determined at 700 nm. The ferric reducing power of ACPs was evaluated by the equation as described by Shang *et al*.^7^.

### Statistical analysis

All measurements were repeated in triplicate. The values were subjected to one-way ANOVA using SPSS software (19.0). Significant difference of the results was evaluated by Duncan post hoc test, and the differences were regarded as significant and highly significant when the *P*-values were less than 0.05 and 0.01, respectively. The curve estimation procedure was used to estimate the influence of ACPs on the antioxidant capacities.

### Data availability

All data generated or analyzed during this study are included in this published article.

## Electronic supplementary material

Supporting Information S1 Dataset

Supporting Information S2 Dataset

Supporting Information S3 Dataset

Supplementary Information
