# Supplementary material for: Extraction condition optimization and effects of drying methods on physicochemical properties and antioxidant activities of polysaccharides from Astragalus cicer L
Source: Sci Rep. 2018 Feb 20;8:3359. doi: 10.1038/s41598-018-21295-z (PMC5820361; doi:10.1038/s41598-018-21295-z)
Supplement: Supplementary file 4 — Supplementary Information [file 41598_2018_21295_MOESM4_ESM.pdf]

Extraction condition optimization and effects of drying methods on physicochemical properties and antioxidant activities of polysaccharides from *Astragalus cicer* L.

Hongmei Shang[1,2,3], Menghan Wang[1], Ran Li[1], Mengying Duan[1], Hongxin Wu[4], Haizhu Zhou [1,2*]

[1]College of Animal Science and Technology, Jilin Agricultural University, Changchun 130118, China. [2]Key Laboratory of Animal Nutrition and Feed Science of Jilin Province, Changchun 130118, China. [3]Key Laboratory of Animal Production, Product Quality and Security, Ministry of Education, Changchun 130118, China. [4]Grassland Research Institute of CAAS, Hohhot 010010, China.

Correspondence and requests for materials should be addressed to H.-Z.Z. (email: zhouhaizhu@jlau.edu.cn).

Supplementary Information

S1 Dataset: Molecular weight distribution of HD-ACPs.

S2 Dataset: Molecular weight distribution of VD-ACPs.

S3 Dataset: Molecular weight distribution of FD-ACPs.
